# The Mechanical Properties of *in Situ* Canine Skeletal Muscle

**DOI:** 10.3389/fphys.2022.862189

**Published:** 2022-06-06

**Authors:** P. D. Allen, J. K. Barclay

**Affiliations:** Department of Physiology, School of Medicine, University of Florida, Gainesville, FL, United States

**Keywords:** muscle, mechanical properties, length tension, force velocity relationship, series elastic (stress: strain)

## Abstract

This study was undertaken to determine if fiber arrangement was responsible for differences in the whole muscle mechanical properties. Experiments were carried out *in situ* in blood perfused dog skeletal muscles at approximately normal body temperature between 36° and 38°C. The following mechanical relationships were studied using a pneumatic muscle lever to measure Tension (P), length (L) and dP/dt**:** and dL/dt with a high frequency oscillograph (500–1000 Hz): 1.) Length:Tension; 2.) Force:Velocity; and 3.) Stress:Strain of Series Elastic. Electron microscopy and fiber typing were done as adjunctive studies. Muscles were stimulated by direct nerve stimulation with 0.1msec stimuli at a rate of 1 impulse per second for twitch contractions, or in 200 msec bursts of 100 Hz 0.1 msec stimuli for brief tetanic contractions. The pennate short fibered gastrocnemius plantaris developed 1.0 kg/g of tension during brief tetanic stimulation, at optimal length (Lo) with full stimulus voltage, while the parallel long fibered semitendinosus developed 0.5 kg/g under the same conditions. The Length:Tension relationship for these two muscles was qualitatively similar but quantitatively different. The Force:Velocity relationship (ΔL/L_0_ vs. P/P_0_) for both muscles were also qualitatively similar and could be described by the previously proposed rectangular hyperbola but a better predicted fit to the observed data could be produced by adding a descending exponential function to the rectangular hyperbola. Unlike previous studies, the Stress:Strain properties of the series elastic component measured by quick release (ΔL/Li vs. ΔP/Po) were linear and gastrocnemius was 25 per cent higher than the semitendinosus. Overall, both muscles were found to have mechanical properties that differed little from the previously reported literature for amphibian, cardiac and small mammalian muscles studied by others *in vitro*. The major differences that we found were in the shapes of the force:velocity curve of the contractile component, and the Stress:Strain curve of series elastic component. Equations and explanations for these differences are devised and presented.

## Introduction

The study of the physiology of skeletal muscle has been dominated by experimentation using muscles of small animals or amphibians *in vitro*. An interesting offshoot began in 1927 when Himwich and Castle described an isolated dog skeletal muscle preparation using the gastrocnemius-plantaris muscle group (Himwich, 1927). The most significant aspect of their preparation was the intact circulation to the muscle. Their descriptions of the preparation were used by Stainsby et al. (Fales et al., 1956) and established the foundation for much of the experimentation on muscle function and exercise-relevant work from the Stainsby’s laboratory at the University of Florida (Fales et al., 1956; Stainsby, 1964; Stainsby and Welch, 1966; Stainsby, 1970; Stainsby and Barclay, 1970; Barclay et al., 1974; Lambert et al., 1979; Barclay and Stainsby, 2004) (for other relevant work search Stainsby WN on PubMed).

Two other dog muscles have been considered for experimentation in this laboratory. They were the dog gracilis and the dog semitendinosus. The isolation of the gracilis circulation was very difficult to accomplish and the preparation was judged to be not practical. On the other hand, the semitendinosus proved to be very useful because of its parallel fibered structure even though aspects of the vascular system and the neural connection were more difficult to isolate than in the gastocnemius-plantaris (Stainsby and Barclay, 1971).

These two preparations with intact circulations allowed maximum freedom in designing experiments. The level of blood flow could be controlled with pump perfusion. Arterial gas concentrations could be modified by changing the gas mixture attached to a ventilator. Arterial concentrations of other substances could be modified by infusing the compound into the arterial line. The initial conditions of muscle length, load, and stimulation parameters could be set as could muscle temperature.

Two problem areas were the stimulation parameters and the fiber composition. The issue with stimulation is that the voltages used were such that all motor neurons in the nerve would be activated with each stimulus resulting in all of the muscle fibers being activated at the same time independent of the fiber type. The modern fiber typing system has become more complex but the original observations fiber typing dog muscle still hold (Maxwell et al., 1977) *i.e.* canine muscle fibers are all fatigue resistant but have different percentages of slow and fast fibers (gastrocnemius with 55% slow and 45% fast while semitendinosus is 38% slow and 62% fast). Are these differences in muscle composition enough to observe differences in function?

The thing that is missing from a discussion of the preparations of dog skeletal muscle with intact circulation is a systematic comparison of the mechanical characteristics of the two preparations. To address this, we tested the hypothesis that the mechanical characteristics of the parallel fibered semitendinosus would be the same as those of the gastrocnemius-plantaris group which has a bipennate and a spiral component by asking the following questions: 1) Are there any significant differences in mechanical properties observed at 37°C in relation to: Length:tension, Force:Velocity and the Series Elastic component (Stress:Strain) between the long parallel fibered semitendinosus and the short fibered gastrocnemius-plantaris where the fibers in a pennate spiral arrangement around a central tendon. and 2) Are the mechanical properties of these canine muscles significantly different from other mammalian skeletal muscles examined *in vitro* or *in situ* at body or lower temperature. These studies were carried out between 1970 and 1973 to provide clarity of these muscle’s mechanical properties for later studies using these muscles as a model.

## Methods

### Experimental Animals

54 mongrel dogs of either sex weighing 8–12 kg were used as experimental animals as approved by the IACUC at the University of Florida, College of Medicine. All animals were kept in runs and observed for 3 weeks prior to the experiment and passed requirements for good health set by the animal department. All had normal white blood cell count, hematocrit and sedimentation rate. The dogs were not screened for heartworms or intestinal parasites. For initial anesthesia each received 30 mg of sodium pentobarbital per kilogram of body weight intravenously (IV). This was supplemented as needed during the experiment with additional doses of IV pentobarbital. The left leg was shaved and the muscle used was cleared from other tissues and attached to a pneumatic myograph, which is described later in detail, used for measurements of mechanical activity (Fales et al., 1958). The animals were humanely euthanized under anesthesia at the end of the experiment with pentobarbital and IV potassium chloride.

### Preparation Used

**FIGURE 1:**
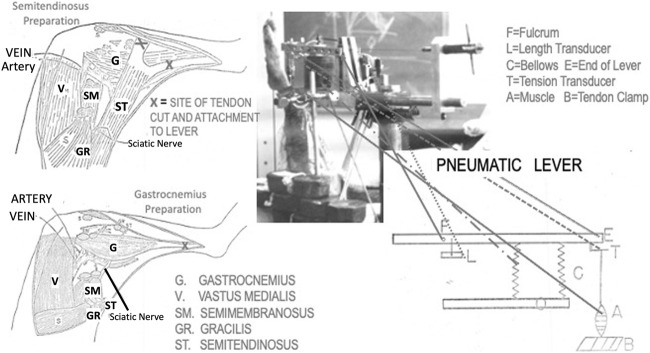
Artist’s depictions of the two muscle preparations used in this research and the pneumatic lever used to make the measurements. Also, at the top right there is a photograph of the lever system in use for a semitendinosus preparation.

The perfused denervated dog gastrocnemius (Fales et al., 1956) and semitendinosus (Stainsby and Barclay, 1971) with isolated intact circulation is a unique model because unlike almost all other preparations it gives the experimenter the ability to change almost all variables in muscle physiology except recruitment of motor units which all fire at once when the nerve is stimulated directly. Temperature, initial length, load (preload and afterload), stimulation frequency and determination of the elastic properties can all controlled independently (See Figure 1).

### Fiber Typing

Fresh biopsy samples both gastrocnemius-plantaris and semitendinosus muscles were quick frozen and liquid nitrogen and placed on frozen section chucks for sectioning. Half micron sections we’re stained for myofibrillar ATPase pH 9.4 and succinate dehydrogenase (27). These sections were then viewed on an upright microscope, photographs were taken, and the fibers typed according to their staining properties.

### Electron Microscopy

After completing a series of dynamic length tension curves, the blood vessels to the muscle were isolated and the muscle perfused *in situ*, with fixation solutions while still attached to the lever so that fixation would be done a known initial length. Each muscle studied was fixed at a different initial length that was used to measure isometric tension for the Length:Tension Curve. This was done so that a sarcomere length-tension relationship could be ascertained relative to the active length tension curve. After fixation, initial embedding was done using a one-to-one ratio propylene oxide and araldite-epon mixture for 2 h and then followed by 12 h in a one to two mixture. The specimens were then cut into still smaller pieces and placed longitudinally in the tips of small plastic capsules and covered with 100% araldite-epon mixture. This was allowed to polymerize for 3 days at 60°C. At first 5 µ thick sections were made to ascertain fiber orientation after which the blocks were trimmed and thin sectioned (∼400–600 Å) and placed on 300 mesh coated copper grids. These were viewed in a Hitachi 11 TC electron microscope. Photographs were made on Kodak high resolution electron sensitive plates and printed on Brovira #6 photographic paper. Sarcomere lengths were determined by measuring the distance between the midpoints of two adjacent Z dash lines. I band and A band widths were also measured.

### Muscle Isolation

Either the semitendinosus or the gastrocnemius plantaris was used as the experimental muscle. Only one muscle was used for each animal. Isolation of the muscle was performed following the techniques of Stainsby and Barclay (Stainsby, 1970; Stainsby and Barclay, 1972). Briefly, the semitendinosus was isolated through a wide anterior medial incision and was cleared of its connective tissue attachments to the skin, being careful to preserve the blood supply (see Figure 1). Next, its two tendons were cut from their insertions and placed into a lightweight aluminum tendon clamp, the leg was then turned over and a posterior incision made, the nerve to the semitendinosus was exposed carefully isolated, ligated at the central end, cut between the ties and the distal end laid down on a silver wire stimulus electrode imbedded in a tubular dental acrylic nerve holder. The distal cut end of the nerve was loosely tied in place and the holder was filled with isotonic saline and the ends were sealed with silastic grease. The posterior wound was then closed around the electrode leads with skin clips and the leg turned over and a bone nail was placed into the bony pelvis parallel to the leg and perpendicular to the pelvis and attached to a strut which butted against the tip of the myograph support. The tendon clamp was attached to the myograph (See Figure 1). The strut and bone nails provided mechanical stability by preventing the muscle from pulling the animal up towards the lever. The gastrocnemius plantaris was isolated similarly using an anterio-medial incision (See Figure 1). Its tendon was removed from its insertion to the calcaneus and was placed in the tendon clamp. The proximal sciatic nerve was isolated and treated in the same fashion as the semitendinosus nerve. Two bone nails were placed into the femur at 90° to the pull of the muscle these were clamped to the myograph stand with struts which screwed into bone nails. A large strut was placed parallel to the pull of the muscle between the bone nail and the myograph preventing both lateral and rotational movement as well as providing the vertical stability needed because of the large tensions developed by this muscle. Again, the tendon clamp was attached to the myograph, and measurements made.

### Myograph

The isotonic and isometric lever system used for these experiments was a modification of the pneumatic isotonic lever system describe by Fales (Fales et al., 1958). (See Figure 1). The muscle exerts its force against the air in the bellows. To maintain the bellows air pressure nearly constant during the compression phase a surge chamber 1,000 times the volume of the bellows was connected to the bellows by a 2″ bore tube. Further, the range of complete excursion made by the bellows during the maximum contraction produced a volume change of less than 1/3 of the volume in the bellows. Therefore, a contraction altered the gas volumes by a maximum ratio of only 1–3000 making pressure quite constant in the bellows throughout the contraction. The inertial load of the air column in the tube was very small and when added to that of all the mechanical parts of the lever, the total inertia is equal to about an 86 g mass at the end of the lever which is approximately 1.9 g/g of either muscle. The stiffness of the bellows was overcome by a spring adjusted to be exactly equal and opposite to the stiffness of the bellows as described in detail by Fales et al. (1958). An aluminum rod with regular indentations was used to connect the muscle clamp to the force transducer on the lever. The distance between the indentations in the rod was 6 mm. A stainless-steel wire was used to connect the tendon clamp to the rod.

Muscle shortening was measured using a Statham strain gauge linear displacement transducer placed about 8 mm forward of the pivot point of the lever. Its output was linear over 4.5 cm of travel which was the maximum allowed by the lever. A small range (1 cm) of non-linearity near the top of the travel of the lever was illuminated by a stop above the after-load screw which was always left in place. Tension (P) was measured using two bonded semiconductor strain gauges placed on a 1/4 in thick by 1/2 in wide by 1 in long aluminum bar which ended in the clamp for the muscle attachment rod. The compliance of the rod and the transducer and lever system was approximately 10^–4^ cm/kg tension development. This allowed a maximum movement that was less than 1% a muscle rest length (Lr) at the maximum isometric tension development. Velocity and the rate of tension development during the isometric contraction phase were measured by taking the amplified signal from the length and tension transducers respectively and placing it through a R/C op-amp differentiated network. The addition differential R/C op amp circuits constructed to have a resistance and time constant for maximum sensitivity and linearity over the range studied (0–100 Hz natural frequency). For quick release to measure the series elastic component, a delay circuit was built which powered a solenoid near the end of its travel to allow maximum acceleration of a stop which held the lever. 100 msec after initiation of a contraction, the solenoid was activated and the stop was pulled, releasing the lever. This allowed an almost instantaneous change (4.0 msec) in the length of the muscle from its rest isometric length to the new isometric length corresponding to the new load.

### Recording Equipment

All recorded signals were conditioned with a Honeywell Accudata 113DC bridge amplifier (Honeywell Inc. Newport TN). Excitation voltage to the position and tension transducers was 4.5 V DC. The output of the amplifier was transmitted to a Honeywell M1650 galvanometer and the light signal from the galvanometer recorded on Kodak 1895 linograph light sensitive paper. The M1650 galvanometers had a frequency response which was linear to 1000 Hz (10X the natural frequency of the event). The paper speed for recording was run between 100 and 2,000 mm/sec. The output of the amplifiers was also monitored on a single beam eight channel oscilloscope. Filters were then placed at a frequency of five times that of the maximum expected requirements (60 Hz for muscle length and tension and 100 Hz for the differential signals) to assure less than 1% error in the actual measurements. The signal to the differentiators was taken after filtering but before it reached the recording galvanometer so the differentiated signal would be in phase with the non-differentiated length and force outputs. In the final version, signals from the length and tension amplifiers were conditioned to 500 Hz and that from the differentiators to 1000 Hz using three pole Butterworth filters at a rate of 4DB/octave. The response was checked with a signal generator using both sine and triangular wave forms from 1 to 100 Hz for phase and amplitude linearity and was found to be linear and in phase over the entire range. All instruments were calibrated daily with constant input source in (1 cm for length transducers, 4.4 kg for tension transducers). The differentiated output was calibrated by applying a 10 Hz triangular wave signal to the length or tension amplifiers and recording the wave function height, frequency and 1/2 maximum excursion of the square-wave differentiated output.

### Nerve Stimulation

Direct nerve stimulation was done using square pulses of 0.1 Msec duration and 8 V amplitude for the normal voltage experiments and for the reduced voltage experiments from the amplitude of the voltage used was 0.12–0.30 V which reduced the force development at L_0_ to 0.5–0.8 (mean 0.65) P_0_. The stimuli were produced by using a single or two S5 grass stimulators coupled in series. Two types of experiments were done using the same stimulus pulse characteristics. 1. Twitch: one 0.1Msec impulse per second. Brief Tetanic: 200 msec duration trains of 0.1 Msec 100 Hz impulses at a rate of 12 per minute. This rate and duration of stimulation for tetanic contractions was chosen empirically as the lowest frequency of stimulation that resulted in smooth fused tetanic contractions with no decline in force over time. At the commencement of the experiment or after any change in experimental conditions, for example change in length, stimulus voltage or load, a minimum of 10 brief tetanic or 20 twitch contractions were allowed to pass unrecorded, being observed only on the oscilloscope to allow for the staircase potentiation effect (Hamada et al., 1985; Peyton and Lowe, 2022) when quiet muscles are first stimulated. After this the data points were gathered for analysis. This procedure was used to reduce errors from changes in contractility due to “staircase effects” because of the changes in load or length.

### Experimental Protocols

#### Length:Tension Relationship

**FIGURE 2:**
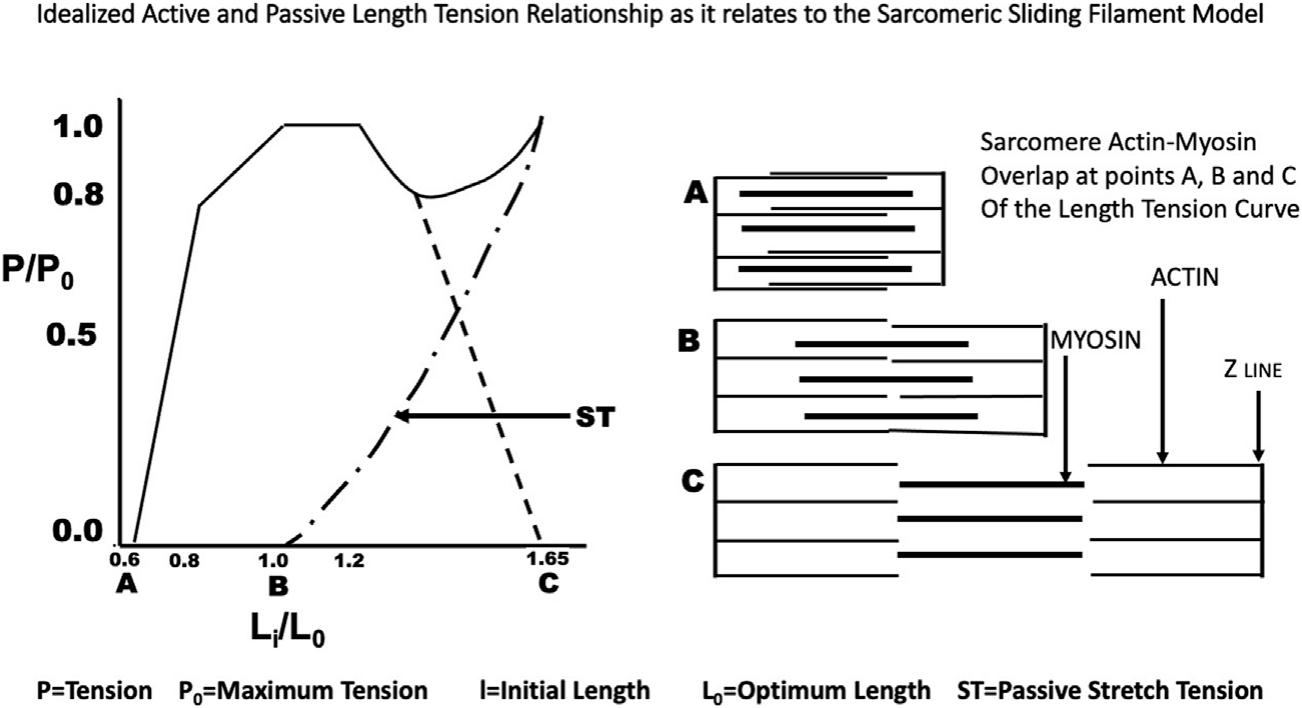
The idealized Length Tension curve as it relates to the overlap of actin and myosin in the sarcomere as described by Gordon, Huxley and Julian (Gordon et al., 1966).

Each muscle studied had isometric length:tension relationships measured for brief tetanic contractions. This was done at intervals throughout the experiment to establish the viability and stability of the preparation and the position of optimum length. Twitch length:tension curves were also done on some semitendinosus muscles. Twitch length:tension curves on the gastrocnemius plantaris was reported previously (Stainsby, 1970). To establish the length tension relationship, L_i_ was increased in a stepwise fashion from L_r_, and developed tension was measured at each rest length. Actual measured developed tension (P) was then divided by the tension developed at optimum length (P/P_0_) and was plotted against initial length as a percent of optimum length (L_i_/L_0_). (See Figure 2).

#### Force:Velocity Relationship

Force:velocity curves were done using a protocol very similar to that used by AV Hill (Hill, 1938) in studies of isotonic contractions (see Equation 1 below) where **V** is velocity of contraction, **b** is a constant, **P**_**0**_ is the maximum isometric force generated at the muscles optimum length, **P** is the force generated at any initial length including optimum length and **a** is another constant.

$$\mathrm{V=b}\left(P_{0}-P\right)/\left(\mathrm{P+a}\right)$$ (1)

All previous studies recognized that during isometric contractions that the active force generated by the muscle was related to the initial length of the muscle peaking at its optimum length and then declining, while passive force presumably caused by the resistance of the connective tissue around the muscle, the muscle membrane, and tendon increases. Later Gordon, Huxley and Julian (Gordon et al., 1966) demonstrated that this shape was related to the overlap of actin and myosin in a single sarcomere, the basic unit of muscle (See Figure 2). Our studies included measurements both at L_0_ and at reduced length at which P_max_ for that length was equal to 0.5–0.8 (mean 0.65) P_0_ and with full and reduced stimulus voltage as described above. The actual force:velocity curves were produced from data obtained by randomly changing the afterload stepwise upward and downward recording both the tension and the velocity occurring during the isotonic contraction. In the semitendinosus the initial length which had no passive tension varied from 0.65 L_0_–1.12 L_0_. Muscles were not stretched further because of reported permanent side effects found secondary to stretch (Stainsby and Barclay, 1976). In the gastrocnemius plantaris it was not possible to see any developed force without passive tension. The optimum range for developed-passive tension was from 0.87L_0_–1.1L_0_. The lower limit was set because the muscle did not develop any tension below that initial length. Each muscle had its force:velocity relationships studied under the following eight conditions (See Table 1). Conditions 1-4 were twitch contractions and conditions 5-8 were Brief Tetanic contractions. In addition to the difference in stimulation type, the same muscles were stimulated with reduced stimulus voltage or L_i_ was reduced below L_0_. Both the length and stimulus voltage changes were empirically designed for each muscle to reduce V_max_ to approximately ½ of that measured for optimal conditions. Conditions 3 and 7 were done at L_i_ = L_0_, full stimulus voltage, Conditions 1 and 5 were done at L_i_ = L_0_, reduced stimulus voltage, Conditions 2 and 6 were done at L_i_ < L_0_, reduced stimulus voltage, and Conditions 4 and eight were done at L_i_ < L_0_, full stimulus voltage.

**TABLE 1:** Parameters for the least squares fit for Eqs 1A, 2 for semitendinosus (top) and gastrocnemius-plantaris (bottom).

	Condition	Stimulus type	L	Mean V_max_(5)	Mean V_max_(3)	Mean P_o_ Difference(5)	Mean P_o_ Difference(3)	Mean a/p_o_
A	1	TW-RV	=L_o_	0.71±0.00	0.39±0.36	0.001±0.00	0.067±0.043	247.1±154.1
	2	TW-RV	<L_o_	1.43±0.00	1.82±0.33	-0.001±0.00	0.016±0.007	54.81±23.65
	3	TW-FV	=L_o_	4.74±0.00	2.79±1.71	0.00	0.088± 0.024	6.75±6.28
	4	TW-FV	<L_o_	2.68±0.37	2.26±0.30	0.014±0.01	0.01±0.061	119.6±35.24
	5	BT-RV	=L_o_	7.27±6.02	1.47±0.33	0.004±0.007	0.059±0.009	4.03±1.97
	6	BT-RV	<L_o_	1.55±0.33	1.94±0.32	0.003±0.003	0.004±0.003	66.17±42.48
	7	BT-FV	=L_o_	2.05±0.59	2.72±0.88	0.03±0.027	0.218±0.079	1.52±0.71
	8	BT-FV	<L_o_	1.58±0.38	1.53±0.18	0.01±0.005	0.155±0.059	117.56±95.75
Semitendinosus ↑ FORCE: Velocity Fits to 3 Parameter and 5 Parameter models ↓ Gastrocnemius-plantaris								
B	1	TW-RV	=L_o_	10.5±3.75	15.9±8.99	-0.004±0.004	0.021±0.031	2.8±1.76
	2	TW-RV	<L_o_	5.5±1.05	5.1±0.42	-0.005±0.004	-0.021±0.056	137.2±21.99
	3	TW-FV	=L_o_	9.9±1.23	9.8±2.11	0.008±0.016	0.057±0.009	71.1±22.59
	4	TW-FV	<L_o_	6.9±0.69	8.4±0.77	0.013±0.010	0.038±0.008	107.5±25.95
	5	BT-RV	=L_o_	13.5±3.13	11.9±3.41	-0.007±0.007	0.014±0.012	4.1±3.61
	6	BT-RV	<L_o_	8.3±1.71	8.2±0.77	-0.004±0.002	0.055±0.526	34.2±19.62
	7	BT-FV	=L_o_	10.8±1.06	13.5±3.19	-0.007±0.007	0.035±0.011	1.4±0.41
	8	BT-FV	<L_o_	10.7±1.76	8.7±0.43	-0.013±0.017	0.045±0.068	16.1±10.87

Data are shown are stimulus type TW = twitch BT = Brief Tetanic, Stimulus voltage RV = reduced voltage, FV = full voltage, and L_i_ where = L_0_ is L_i_ = L_0_ and <L_0_ is L_i_ < L_0_. The mean V_max_ predicted by Eq. 2 = (5) and Eq. 1A = (3), the difference between observed P_0_ and predicted P_0_ by Eq. 2 = (5) and Eq. 1A = (3), and the Mean observed a/P_0_ ratios.

#### Stress:Strain Relationship—Series Elastic

A series of quick release experiments were done to determine the stress:strain properties of the series elastic component. The method used for this was basically the same as described by Bahler (1967) and Wilkie (1949). This method assumes that the contractile element of the muscle shortens at a constant velocity governed by the force velocity relationship. Immediately after release a contracting muscle’s shortening occurs at a rate above the contractile element velocity due to the series elastic and then shifts to a constant velocity at the rate of the contraction element velocity at the new load and length. This sudden change in length is taken to be due to a change in the length of the series elastic element, corrupted by the viscosity of the muscle and the compliance and mass of the lever system. By plotting the change in length extrapolated to time zero (T_0_) against the change in load, the compliance of the series elastic component can be calculated.

### Statistics

All raw data were taken from the records analyzed and curves fitted with the University of California Biomedical Computing BMD statistics package. Force:Velocity curves were fitted with the 3 and 5 parameter functions using the nonlinear least squares regression analysis (BMDX85) and R values were calculated after obtaining the sums of squares for y with the BMD02D package. For stress:strain of the series elastic component the raw data were fitted using a BMD02R linear regression analysis program. Differences were considered significant if the *p* value was <0.05.

## Results

### Anatomic Properties

#### Gross Anatomy

After fixation muscles were examined before and after partial nitric acid digestion to estimate fiber length and fiber arrangement relevant to the length and long axis of the muscle. Semitendinosus: Mean Length ± SD at L_0_ was 11.0 ± 1.03 cm and mean cross sectional area (CSA) ±SD was 2.95 ± 0.84 cm^2^. The semitendinosus fibers appeared to run parallel to each other throughout the whole length of the muscle. There is a tendinous inscription approximately 1/4 of the total muscle length away from the origin perpendicular to the long axis. The fibers on both sides of the tendinous inscription appeared to be in series with one another. Physically, the muscle could be modeled as an elongated cylinder with constant diameter from origin to the point of insertion. All the muscle fibers functionally run the full length of the muscle. This model was used for the calculation of volume and cross-sectional area. Gastrocnemius Plantaris: Mean muscle length ± SD at L_0_ was 9.91 ± 1.07 cm and mean CSA±SD was 2.85 ± 0.86. In contrast to the semitendinosus, the gastrocnemius plantaris has a very complicated fiber arrangement around more or less parallel internal tendons. The fiber arrangement was diagonal in relationship to the insertion tendon with a pennation angle of ∼20°. Because of the great variability within the muscle from place to place, fiber lengths were approximately 1/3 to 1/4 of the rest length of the whole muscle.

#### Ultrastructure

**FIGURE 3:**
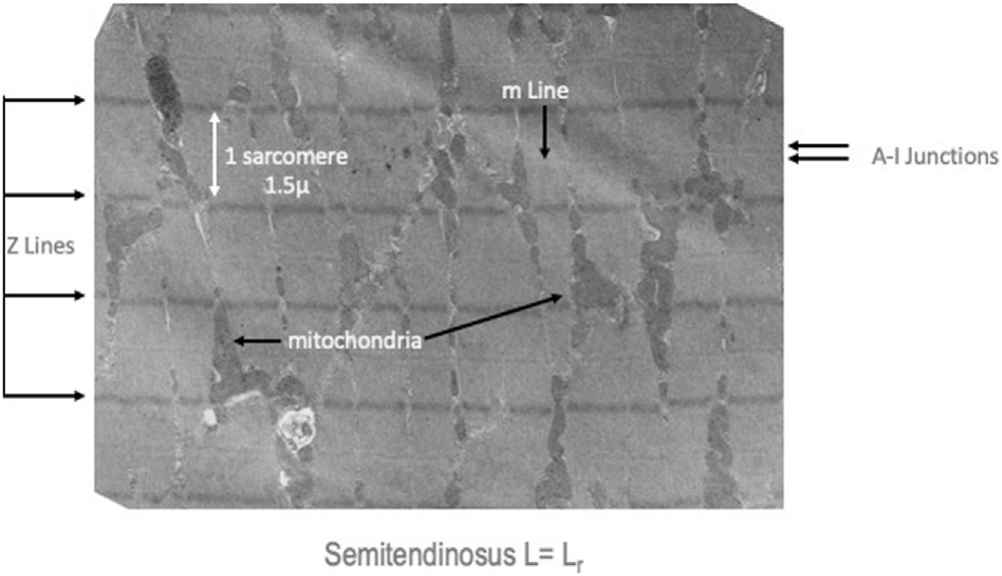
Medium resolution electron micrograph of a semitendinosus muscle at L_r_. The Z lines which demonstrate the distance between sarcomeres, the A-I junctions which are the region where the sarcoplasmic reticulum and t-tubules (invaginations of the surface membrane to make excitation contraction coupling more uniform) are located and indicators of the many mitochondria in these high oxidative muscles.

**FIGURE 4:**
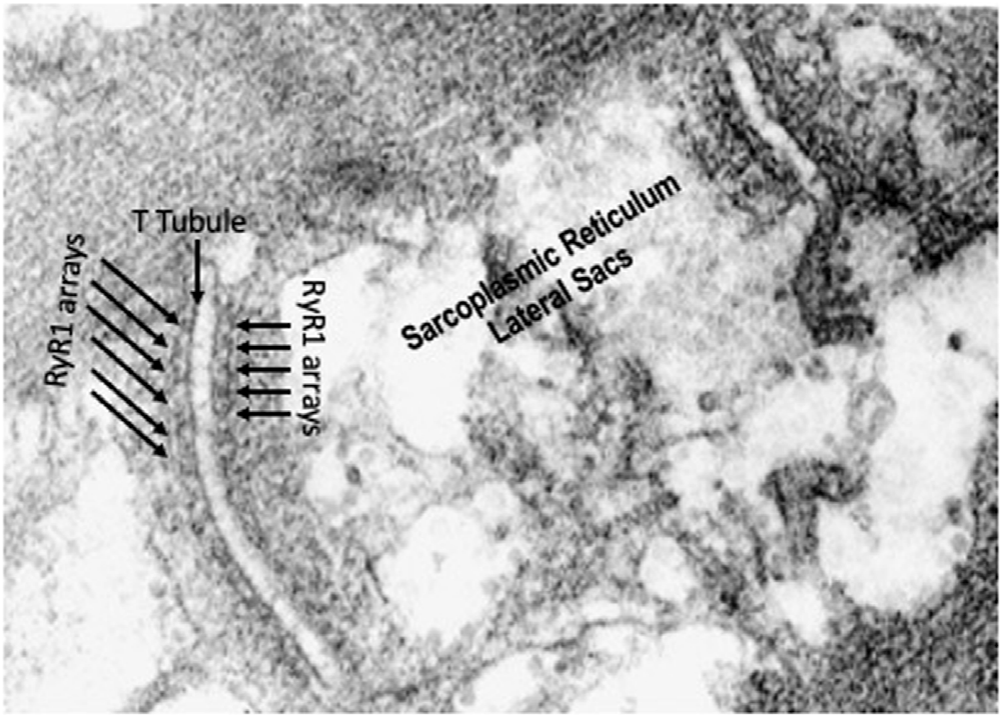
High resolution electron micrograph showing the triads made up of t-tubules/lateral sacs of the sarcoplasmic reticulum and ryanodine receptor arrays on both sides of the t-tubule.

**FIGURE 5:**
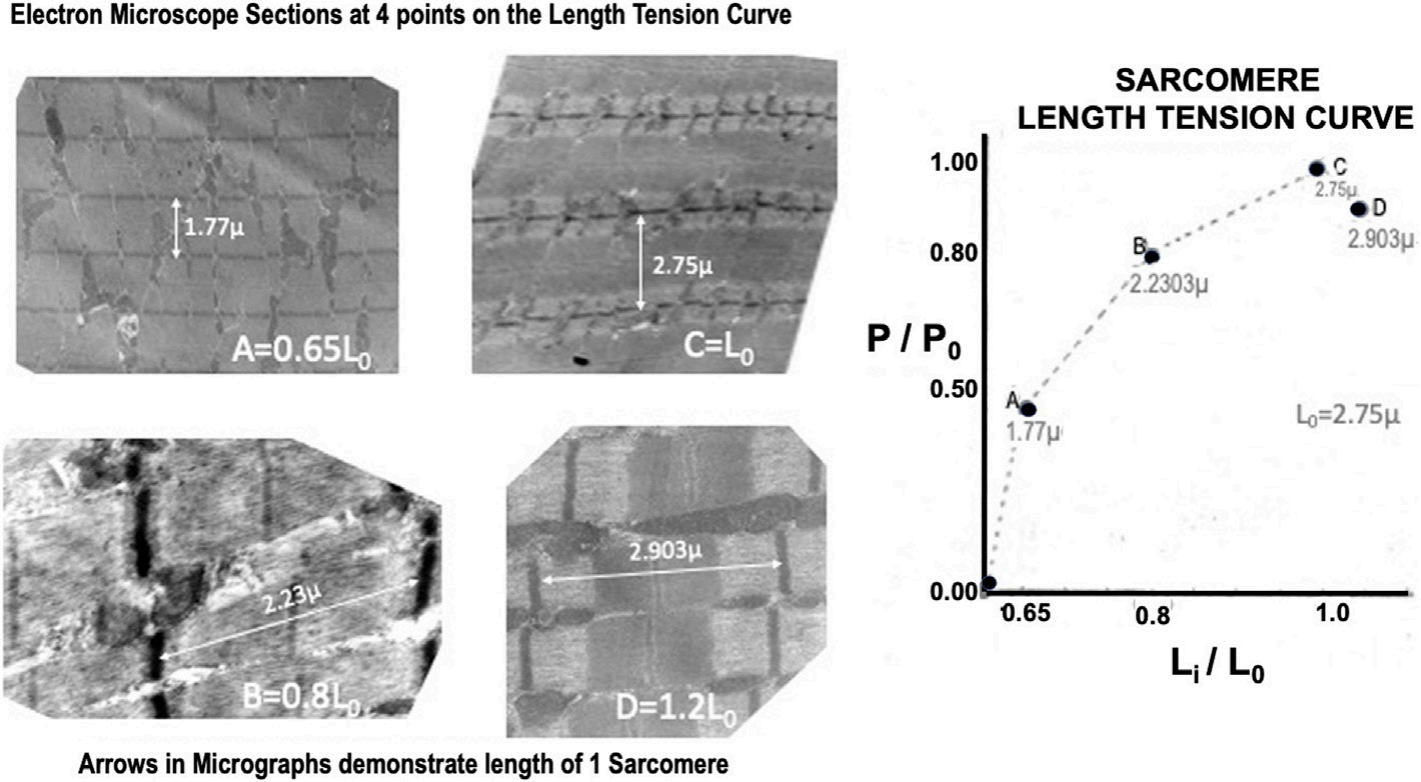
High resolution electron micrographs confirming the sarcomere length:tension relationship. The micrographs on the left are from four muscles that were fixed at different positions on their experimental length: tension relationship.

Electron micrographs made from some of these muscles demonstrated the characteristic picture of an oxidative skeletal muscle with many mitochondria present (Figure 3). The muscles have a prominent triads with the T tubule (the invagination of the surface membrane that permits smooth activation of the contractile components when the nerve is stimulated) located at the A-I junction surrounded on both sides by the lateral sacs of the sarcoplasmic reticulum (see Figure 4). The average sarcomere length of unstimulated semitendinosus at L_0_ was 2.8 microns. The functional length tension curve correlated with sarcomere lengths from electron micrographs as shown in Figure 5 with corresponding EM microscopic sections in which the length of a single sarcomere was measured directly. Figure 3 shows the sarcomere length for gastrocnemius plantaris at length L_i_ =L_r_ where rest tension equals 0. It can be seen here that this muscle produces no force at this length in that it does not reach the minimum functional length needed for contraction when no static preload is applied. When preload was applied the length of the fibers and the length tension curve correlated well with the sliding filament model.

#### Fiber Typing

**FIGURE 6:**
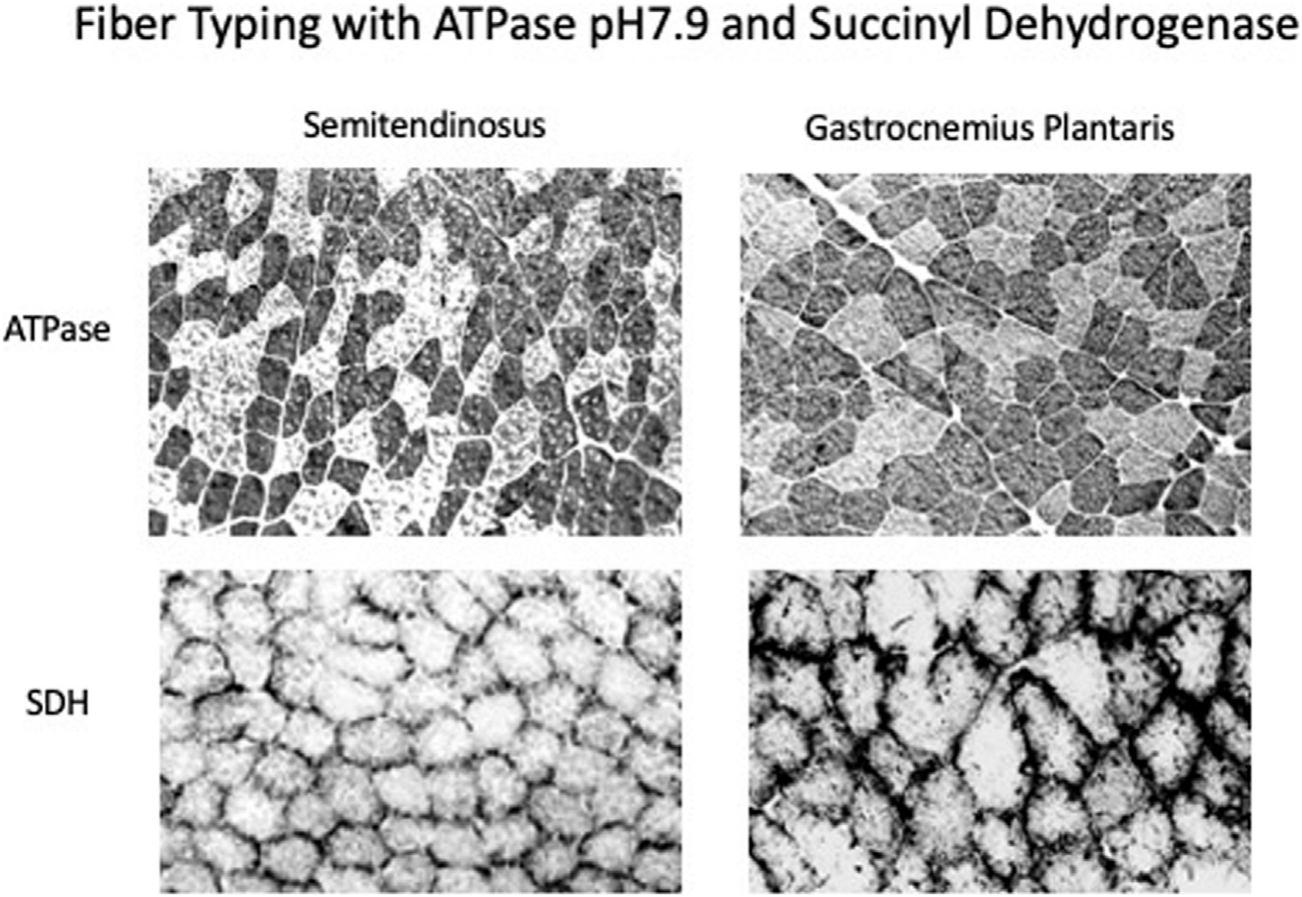
Immunostaining of muscle sections with ATPase, pH7.9 which measures the potential speed of the muscle and succinate dehydrogenase which measures the oxidative capacity. Both the semitendinosus and gastrocnemius plantaris have a mosaic of fast and slow high oxidative fibers.

Both muscles displayed a mosaic pattern when stained for actomyosin ATPase pH 9.6 and uniform staining for SDH showing that they were made up of Fast- and Slow-High Oxidative fibers (see Figure 6). There were no fibers in either muscle that had high staining for actomyosin ATPase and low staining for SDH which is characteristic of classical Fast-Low Oxidative fibers.

### Length Tension Relationship

#### Semitendinosus

**FIGURE 7:**
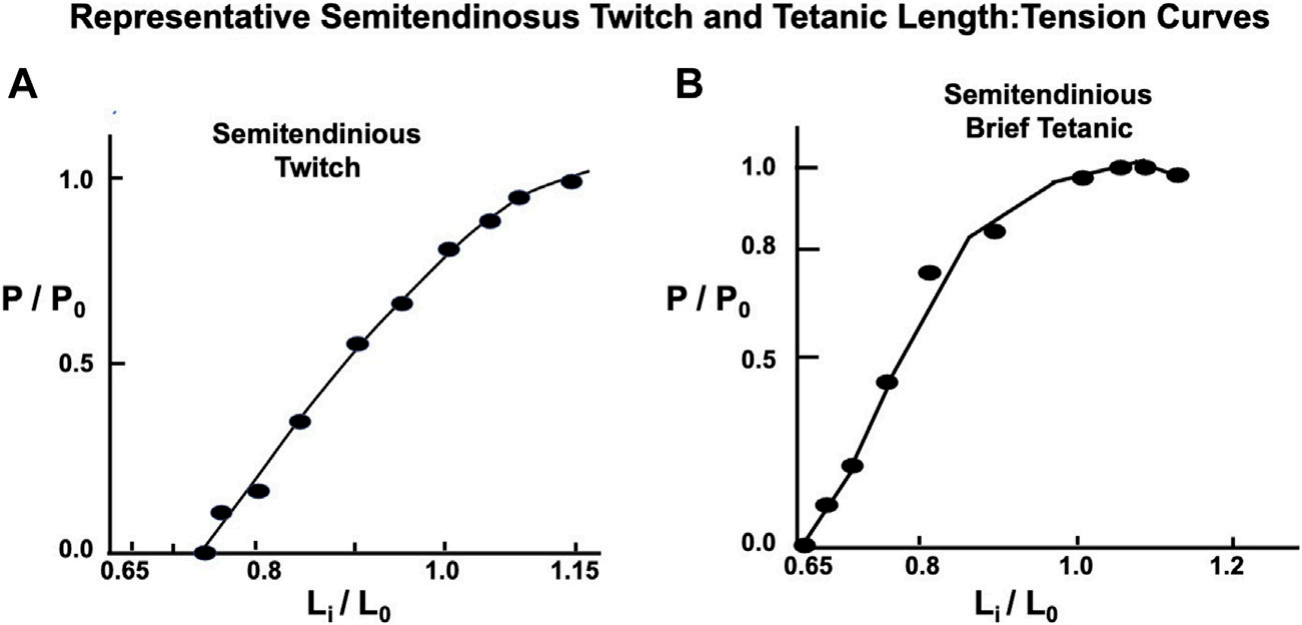
Representative semitendinosus Length: Tension curves with both twitch and brief tetanic contractions. Note in **(A)** L_i_ is located at a longer length as the L_i_ in **(B)** for the brief tetanic contractions, and the twitch curve does not flatten as the muscle is stretched to L_0_ and beyond, as is the case with the classic curve seen in Figure 1B and the brief tetanic length:tension curve from the same muscle in **(B)**.

Twitch length:tension curves were done on 12 animals. A representative twitch length:tension curve appears to be an approximately exponential function crossing the tension axis at L_i_ = 0.68 L_0_ (see Figure 7). The curve peaks at approximately 1.12 L_0_ and continues as a plateau to 1.2 L_0_. Brief Tetanic length:tension curves were done on 16 animals. A representative length:tension curve is displayed in Figure 5. Tension development rises sharply from zero at L_r_=L_i_=0.60–0.65 L_0_ and rises rapidly to approximately L_i_ equal 0.8 L_0_. The curve then flattens from L_i_ equal 0.8 L_0_ to L_i_ = L_0_ after which there is a gradual decline in developed tension which begins between L_i_ = L_0_ and L_i_ = 1.12 L_0_. No experiments were carried out beyond L_i_ = 1.2 L_0_ to prevent muscle damage (Hamada et al., 1985). In the semitendinosus *in situ* L_r_ and experimental L_i_ where developed tension starts were approximately the same. Passive stretch beyond L_i_ = L_0_ produced a small resting tension coupled with a decrease in active developed tension. Electron micrographs for 4 points on the curve in Figure 5 demonstrate the sarcomere lengths at different lengths expressed as L_i_/L_0_. Twitch-brief tetanic ratio under conditions where there is optimum tension development was 0.443.

#### Gastrocnemius-Plantaris

**FIGURE 8:**
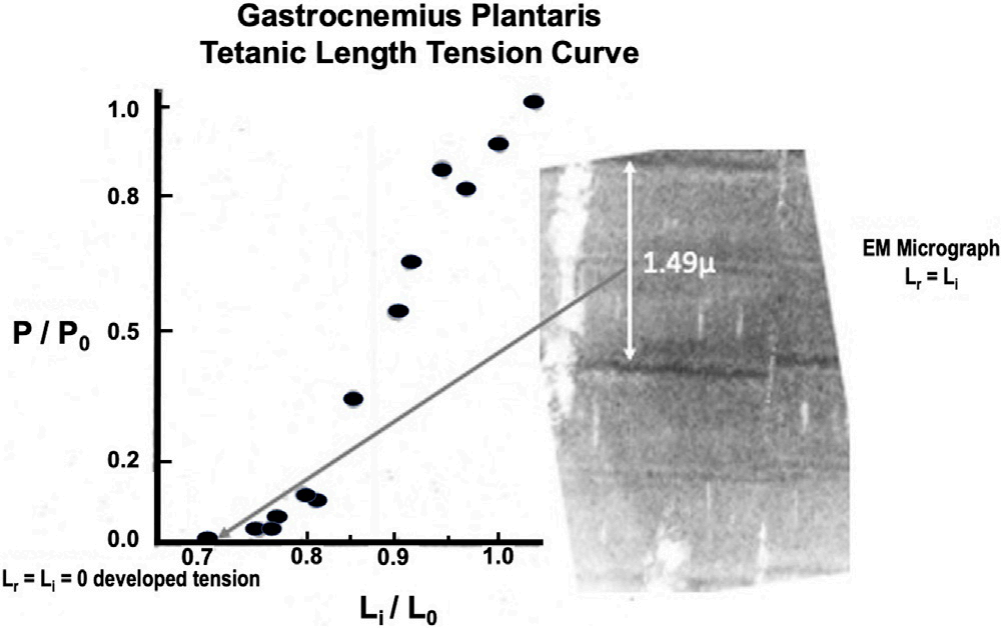
Representative brief tetanic gastrocnemius length tension curve. The electron micrograph on the right was from a muscle fixed at L_r_. Note that there is almost complete overlap of the actin and myosin myofilaments in the sarcomere so that force generation is impossible.

Twitch length: tension curves have been described previously (Stainsby, 1970). Brief Tetanic length:tension curves were done on 16 animals. A representative length:tension curve is displayed in Figure 8. Zero developed tension extrapolates to a whole muscle L_r_=L_i_ between 0.8 and 0.9 L_0_ and requires some passive load to reach this length. This is because the gastrocnemius plantaris has short fiber length and diagonal fiber orientation around a central tendon running parallel through the muscle. At L_r_ the 1.49 µ sarcomere arrangement, as shown in accompanying EM micrograph (see Figure 8) will not allow tension development because there is full overlap of the actin and myosin filaments. Tension increases rapidly to P_0_ at L_i_ = L_0_ and remains constant to from there L_i_ = 1.05 L_0_. Unlike the parallel fibered semitendinosus, L_r_ in the gastrocnemius-plantaris does not approximate L_i_ when tension development begins. This constitutes a major difference between the two muscles along with the fact that the static length tension relationship is shifted far to the left of L_0_ in the gastrocnemius-plantaris compared to the semitendinosus (data not shown).

### Force Velocity Relationship

Before the data are presented for the force:velocity relationship a brief description of the terms and methods of analysis are made to clarify the data which will follow. The force:velocity relationship of an active skeletal muscle has been said to follow the classical relationship first described by Hill (1938) as described the methods section:

$$\mathrm{V=b}\left(P_{0}-P\right)\left(\mathrm{P+a}\right)$$ (B1)

In Eq. B1, P, V and P_0_ are all measured experimentally. **a** and **b** are constants chosen to give the best fit of the equation to a series of observed values of velocity and force. The constant “**a**” has as its dimensions force and the constant “**b**” has the dimensions of velocity. While **a** has the dimensions of force it is most often used in the expression **a**/P_0_ which is dimensionless and has been proposed to be stable over a wide range of muscle lengths and temperatures (Hill, 1938). While constant **b** has the dimensions of velocity as is most often expressed as **b**/L_0_ has the dimensions of seconds^−1^. Hill (1938) states that this ratio may vary enormously according to the functional role and size of the muscle. This relationship however can only hold true when L_i_ = L_0_. If other rest lengths are to be used, by definition, P_0_ must be changed to the maximum isometric tension at the new initial length according to the active length tension curve.

#### Semitendinosus

**FIGURE 9:**
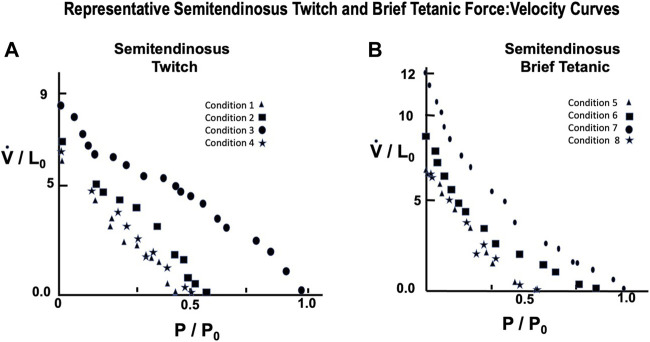
Representative semitendinosus Force:Velocity measurements for twitch **(A)** and brief tetanic **(B)** stimulation. Each muscle was done under four different stimulus conditions, as described in the text. Conditions 3 and 7 represent the relationship at L_i_ = L_0_ and full stimulus intensity. The remaining conditions were used to determine the effect of length and/or activations on this relationship. Conditions 1 and 5 were done at L_i_ = L_0_, reduced stimulus voltage, Conditions 2 and 6 were done at L_i_ < L_0_, reduced stimulus voltage, and Conditions 4 and 8 were done at L_i_ < L_0_, full stimulus voltage.

A representative example taken from a series of force:velocity curves from 12 animals for conditions 1 through 4 are seen in Figure 9A (Twitch) and 5 through eight in 9B (Brief Tetanic). Curves for decreased stimulus voltage at L_0_ (1 and 5) were very similar to curves for full stimulus voltage (4 and 8) and reduced starting length. Groups 2 and 6 overall had the lowest V_max_ and P_max_ and conditions 3 and 7 represent the most robust results when there was full voltage and L_i_ = L_0_.

#### Gastrocnemius Plantaris

**FIGURE 10:**
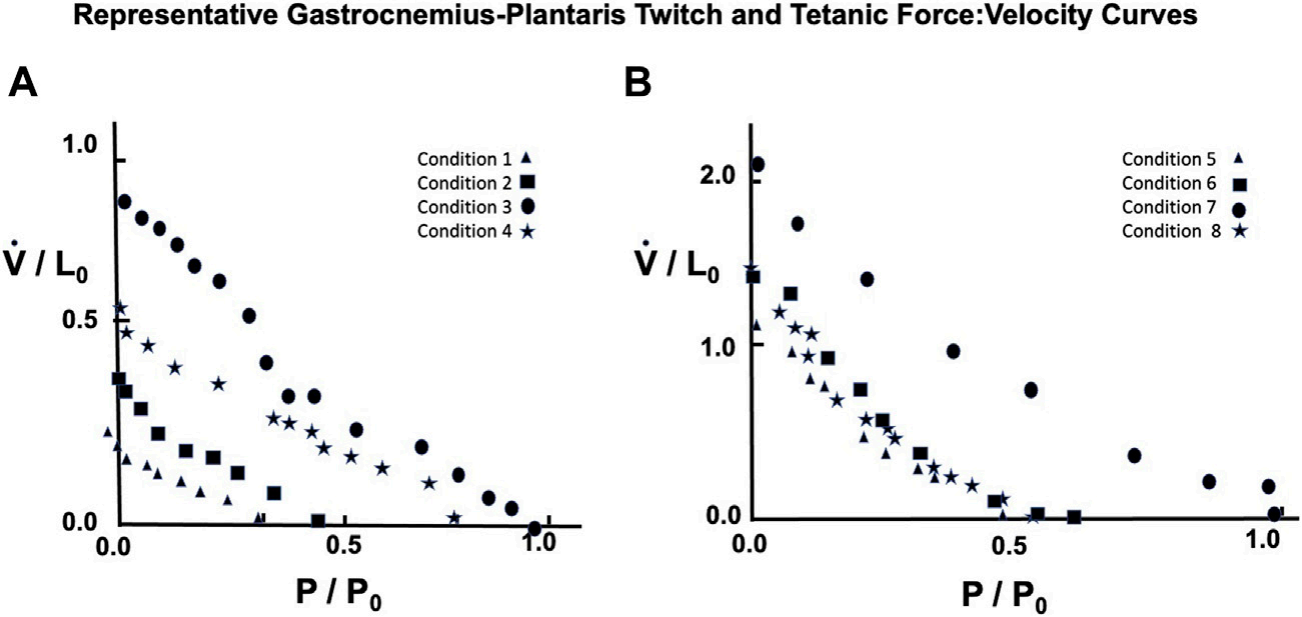
Representative gastrocnemius plantaris Force:Velocity measurements for twitch **(A)** and brief tetanic **(B)** stimulation. Each muscle was done under four different stimulus conditions, as described in the text. Conditions 3 and 7 represent the relationship at L_i_ = L_0_ and full stimulus intensity. The remaining conditions were used to determine the effect of length and/or activations on this relationship. Conditions 1 and 5 were done at L_i_ = L_0_, reduced stimulus voltage, Conditions 2 and 6 were done at L_i_ < L_0_, reduced stimulus voltage, and Conditions 4 and 8 were done at L_i_ < L_0_, full stimulus voltage.

A representative example taken from a series of force:velocity curves from 16 animals for conditions 1 through 4 seen in Figure 10A (Twitch) and 5 through eight in 10B (Brief Tetanic) respectively. When measured in muscle lengths per second compared to the semitendinosus, V_max_ was significantly lower overall but followed the same pattern as Semitentinosus when length and/or stimulus intensity were reduced.

Because semitendinosus is parallel fibered, velocity in muscle lengths per second closely approximates velocity in fiber lengths per second. However, to obtain velocity in fiber lengths per second in the gastrocnemius plantaris to compare it to the semitendinosus, the velocity in muscle lengths must be multiplied by a factor to account for its complicated pennate-spiral fiber arrangement the mean fiber length and the pennation angle which can be measured statically. Since methods to measure neither variable directly where available when these studies were done, we derived this factor from two observations: 1. Gross atomic studies indicated that the individual gastrocnemius plantaris fibers vary in size from 1/3 to 1/4 of gastrocnemius plantaris muscle’s rest length. 2. By extrapolating from the length:tension curve of the semitendinosus, which may be presumed to be comparable to a sarcomeric length tension curve, the difference between L_0_ and L_i_ where tension development is equal to 0 in gastrocnemius (0.8–0.90 L_0_) needs to be multiplied by a factor equal to the difference between these same lengths in semitendinosus. Correcting estimated V_max_ to fiber lengths per second (using the multiple 3.5 for the gastrocnemius plantaris) gave a V_max_ value for gastrocnemius-plantaris which was not significantly different than the semitendinosus and this factor was used as needed for all other comparisons of the two muscles. A summary of the data for both muscles and the mean difference between mean values and predicted values along with the calculated a/P_0_ values are seen in Table 1.

### Series Elastic Component

#### Semitendinosus

**FIGURE 11:**
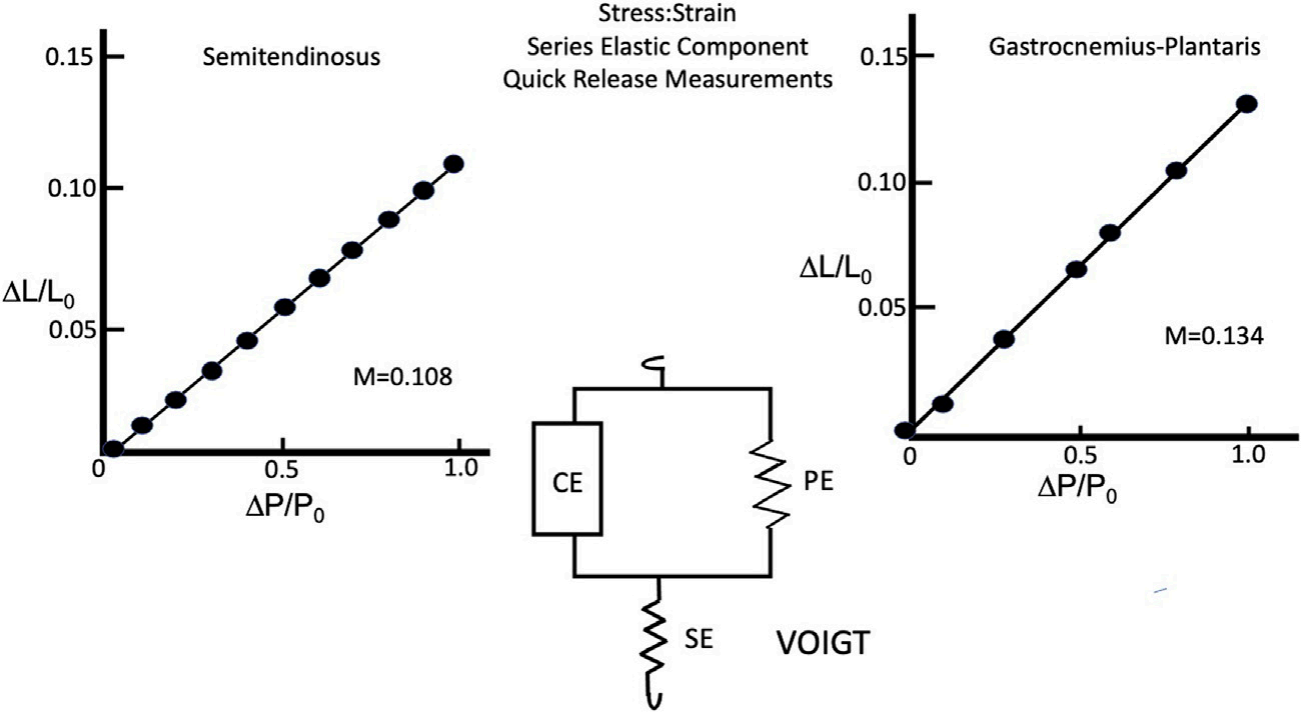
Series Elastic stress:strain relationship of semitendinosus **(A)** and gastrocnemius plantaris **(B)** and the Voigt model (center) describing how the series elastic and parallel elastic interact with the contractile elements.

The composite stress:strain curve for the series elastic component at L_i_ equal to L_0_ and full stimulus voltage for five animals as seen in Figure 11A. The curve of best fit for the data obtained both individually and grouped was a straight line. Extrapolated to P/P_0_ = 1 the series elastic component accounted for 0.108 sarcomere lengths of contractile component shortening under isometric conditions. Changing the initial length and/or the stimulus voltage yielded points that fell on the full stress:strain curve as would be expected for a muscle whose fibers were in series, indicating that series elastic was best represented by the Voigt model (Ford et al., 1977) (see Figure 11). Gastrocnemius Plantaris: The composite stress:strain curve for the series elastic component at L_i_ equal to L_0_ and full stimulus voltage for five animals as seen in Figure 11B. There was more variation in the slope of the line from muscle to muscle in gastrocnemius plantaris than in semitendinosus, but the mean slope is not significantly different than any of the five individual curves, and the line of best fit for the data was a straight line, similar to semitendinosus. Where P/P_0_ equals 1 the contractile element allows 0.134 muscle lengths of contractile component shortening under isometric conditions.

Like semitendinosus the slope of the line for gastrocnemius-plantaris was not altered by changing rest length alone, however while it remained a linear function, its magnitude was significantly increased under isometric conditions when both rest length and stimulus voltage were reduced simultaneously. The difference in the compliance of the series elastic component between the two muscles was not statistically different at L_i_ equal to L_0_ and full stimulus voltage. The gastrocnemius plantaris however, had a significantly higher effective compliance of the series elastic component when L_i_ was < L_0_ and stimulus voltage were reduced.

## Discussion

One of the purposes of this study was to establish whether-or-not the mechanical properties of *in situ* dog skeletal muscle differed from the properties of amphibian and/or small mammalian muscles studied *in vitro*. Commonly used methods of study were employed wherever possible to facilitate comparisons with previous work. The study was divided into four major sections, and each will be discussed in turn: 1) Fiber anatomy and fiber type, 2) length:tension, 3) force:velocity and 4) stress:strain of the series elastic component.

### Fiber Anatomy and Fiber Type

In studying the muscle using lower power electron microscopy on sections from muscles held at L_r_ which prior to fixation, the muscles are rich in mitochondria throughout suggesting that they have a highly oxidative metabolism. The micrographs also show that as expected the SR forms “triads” at the A-I junction with the lateral sacs of the SR being adjacent to the t-tubules. Fiber typing done using standard methods available at the time the experiments were done showed that both the semitendinosus and gastrocnemius-plantaris were made up of a mosaic of High and Low myosin ATPase staining fibers and as predicted by the abundance of mitochondria, all fibers had high SDH staining, indicating that the muscles studied were made up of both Slow (Type I) and Fast (Type II presumably type IIa) high oxidative fibers. This is in concurrence with more recent fiber type data showing that using myosin ATPase staining in dog skeletal muscle has both fast IIa and IIx and slow fibers, all with high oxidative capacity (Amann et al., 1993; Acevedo and Rivero, 2006; Toniolo et al., 2007; Schiaffino and Reggiani, 2011). In addition there is no evidence that we could find that showed that fiber type had any relationship with muscle activation (Schiaffino and Reggiani, 2011) which might have effected mechanical properties.

### Length Tension Relationship

Both the passive length:tension relation of the parallel elastic component and the active length:tension relationship of the contractile component were examined. From these data, a well-defined optimum length (L_0_) was established for maximal isometric tension during brief-tetanic contractions. Studies were not carried out beyond 1.20 L_0_ because attempts to make measurements beyond this length early in the study had resulted in permanent damage to the muscle demonstrated by reduced tension development at optimum length as had been previously observed in other mammalian muscles by Bahler et al. (1968). The exact mechanism for this is unknown as this is not the case for amphibian muscle. From our electron microscopic length:tension study (see Figure 5) it can be seen that the relationship between sarcomere length and active developed tension follow the findings of Gordon et al. (1966) in that the degree of overlap of myofilaments appeared to govern tension development. The sarcomere length at L_0_ is 2.75 microns which is about the same as the sarcomere length at L_0_ measured in frogs and other mammalian muscles studied *in situ* or *in vitro* (Parmley et al., 1970; Gregor et al., 1988).

Our results agree with the length tension relationship for twitch contractions in mammalian muscles studied previously (Close, 1964; Geffen, 1964; Close, 1965; Bahler et al., 1968). The relationship obtained for twitch contractions differs from brief tetanic contractions in two ways, the shape of the length tension curve for twitches is a smoother function, and the apparent optimum length of the muscle is 12% greater for twitches than for brief tetanic contractions. One explanation which has been offered for these differences is that tension development is not only related to the overlap of the thick and thin myofilaments, but also to the fact that the degree of activation of the contractile elements is not complete during a single twitch (Bahler et al., 1967; Rack and Westbury, 1969; Lambert et al., 1979). This relationship between length and activation is not as important for brief tetanic contractions as it is in twitch contractions, because the duration of stimulation is long enough to fully activate the muscle, regardless of length. This theory is unproven and could not be refuted or supported by the present study because no measurements of active state were made.

### Force:Velocity Relationship

All of the force:velocity data collected in these experiments were subjected to nonlinear least squares analysis and the parameters were adjusted to give the curve the best fit. When the force: velocity relationship of the contractile component was studied under optimal conditions, L_i_ = L_0_ with supramaximal stimulus voltage and either twitch or brief tetanic stimulation, the best fit curves showed the relationships illustrated in Figures 9A,B. These data generally conform to the relationship defined by Eq. 1 as suggested by Hill (1938). In previous descriptions of the mechanics of skeletal muscle these constants have either been used at the beginning of the experiment from previous reported constants or have been taken from the first L_i_ = L_0_ F:V curve and held constant throughout the rest of the analysis. In our analyses here however, these values are not held to be constant but rather adjustable parameters to allow the best fit for the equation. Secondly, P_0_ in Eq. 1, which had previously been taken as a constant from an experimental curve, was also allowed to act as parameter “**c**” because there was no justification for that single observed point being more valid than any other observed experimental point. This yielded the equation:

V=b(c-P)/(P+A) (1A)

(see Figure 12).

**FIGURE 12:**
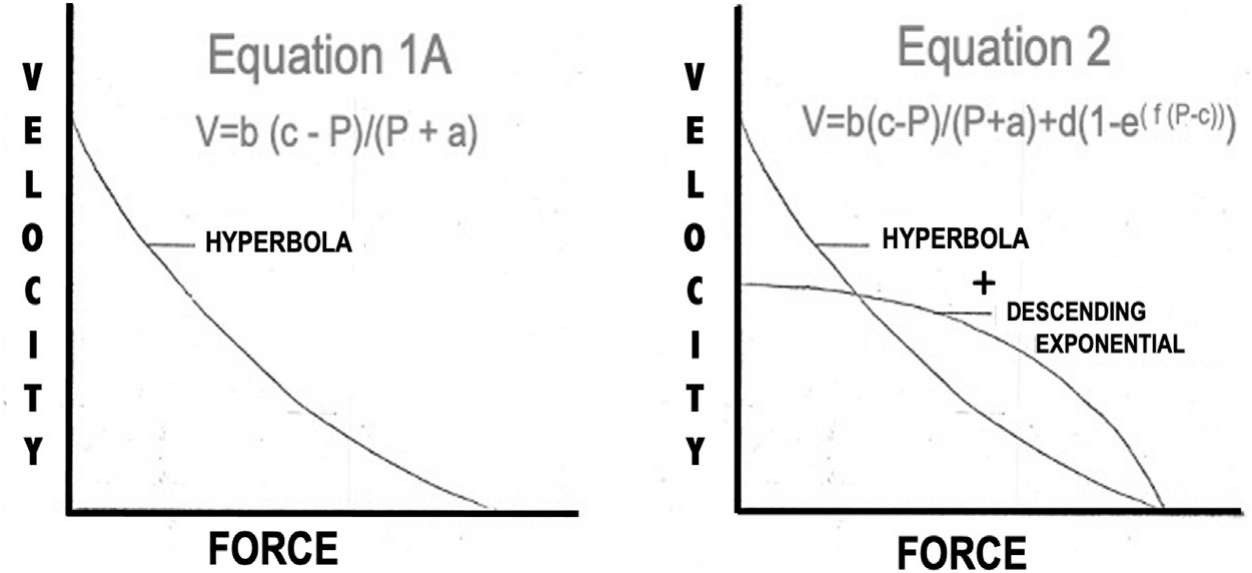
Two models proposed to describe the shape of the Force:Velocity curves of both muscles.

The constants from Eq. 1A obtained by fitting the general equation to the observed data of a single experiment using the above stimulus conditions were: a/P_0_ = 0.63 and b = 2.83. The correlation coefficient between the curve fitted by Eq. 2 and the observed data was 0.9887, *p* < 0.001, indicating that Eq. 2 closely describes these observed data values for the a/P_0_ ratio reported previously from isolated mammalian muscles (0.115–0.60) becoming higher in fast muscles than in slow muscles (Donald et al., 1972; Amann et al., 1993; 83). The speed of the muscles currently studied are relatively fast so that the a/P_0_ ratio would be expected to be at the upper part of the range.

When both semitendinosus and gastrocnemius plantaris muscles were studied under non-optimal conditions (Li ≠L0 and/or twitch contractions) a/P_0_ became larger and more variable ranging in magnitude from 1 to 2.5^.^10^4^ the a/P_0_ ratios indicate the relative concavity of the force velocity curve. Elevated a/P_0_ ratios indicate little curvature, and the force velocity curve is nearly a straight line, but there is no quantitative relationship between shape and the magnitude of the a/P_0_ ratio. The data from contractions done at all conditions demonstrate a wide variation in the quantitative results between different muscles. In contrast, force velocity data from previously reported experiments done under optimal conditions had been more consistent for muscle to muscle (Fenn and Marsh, 1935; Wilkie, 1949; Ritchie, 1954; Rosenblueth and Rubio, 1959a; Close, 1965; Gordon et al., 1966; Lawrence McCrorey and Alpert, 1966; Joyce and Rack, 1969; Parmley et al., 1970; Ameredes et al., 19851992; Perreault et al., 2003; Hakim et al., 2013; Herzog, 2017) Variation in the data obtained among different muscles however, is not unique to the present study. It has been observed previously by Stainsby (1970), Stainsby and Barclay (1971), Stainsby and Barclay (1972) and Fales et al. (1956), who used preparations identical to those used in the present study. The cause of the wide variation of data from muscle to muscle was not determined.

Deliberately decreasing the muscles ability to contract maximally causes changes in the force:velocity relationship other than just altering the a/P_0_ ratio. When L_i_ was reduced below L_0_ the maximum isometric contraction strength was reduced and shortening velocity was reduced at all loads (see Table 1). The mechanism for the reduction in velocity has not been adequately explained but may be due to delayed complete activation of the contractile elements because of reduced L_r_. The present study offers no data on differences in activation at lower lengths as all motor units were activated simultaneously. The reduction seen in predicted V_max_ was much less (2–12%) than the reduction in maximum tension (10–50%). These data concur with other studies in which initial length was reduced (Rosenblueth and Rubio, 1959b; Rosenblueth and Rubio, 1960; Pennycuick, 1964; Joyce and Rack, 1969). Reducing the muscle’s ability to contract maximally by lowering stimulus voltage, thereby reducing the functional cross-sectional area, reduced the maximal isometric tension between 50 and 80% of maximal isometric tension for super maximal stimulation. Associated with the decrease in maximal isometric tension was a smaller reduction and predicted V_max_. The percent change in V_max_ was between 1/3 and 1/5 as great as the corresponding change in tension. If L_i_ is lowered at the same time as stimulus voltage isometric tension is further reduced from that seen at L_0_ for reduced stimulus voltage. This is accompanied by a modest further decrease of predicted V_max_ for sub maximal voltage at L_0_. It appears from the above that the effects of submaximal voltage and reduced initial length are additive if the two conditions are superimposed. Deliberately lowering stimulation voltage to decrease contractility is a condition which is unique to the present study. As a result, these data could not be compared to the work of other investigators.

The force:velocity relationship for twitch contractions at L_0_, full stimulus voltage, appear similar to the force:velocity relationship for a brief tetanic contractions when the contractile state is reduced. Maximal isometric tension development is approximately ½ of that developed during brief tetanic contractions at the same stimulus voltage and rest length. Predicted V_max_ is reduced less than 5% at L_0_ and the percent reduction in V_max_ is a third to a fifth as great has the corresponding reduction in isometric tension development at all initial lengths. It appears that in twitch contractions, as in brief tetanic contractions, the effects of lowering stimulus voltage and reducing rest length are additive. Despite the similarity of changes in force and velocity brought about by reduced stimulus voltage, reduced L_i_ and twitch contractions the mechanisms for the reduction and contractile state among the different conditions may be different. In twitch contractions all the fibers are activated but duration of activation may be so short that the muscle is unable to realize its maximum potential for either tension development or speed of shortening. Alternatively, reduced stimulus voltage reduces isometric tension development by reducing the total number of active contracting fibers and reduces the maximum velocity by adding the mass of the unstimulated fibers to the inertial load which the remaining muscle fibers must accelerate; this in turn reduces velocity. Changing L_i_ above or below L_0_ reduces force and velocity by reducing the degree of overlap between the actin and myosin in the sarcomere. Changing L_i_ reduces the number of active sites which are available for potential crossbridge formation, thereby reducing the potential for tension development and/or shortening.

Two modifications were made in our regression analysis of the force:velocity relationship which differ from previous analyses. (1) P_0_ was made into a third variable parameter, c, and (2) the remaining parameters in this model are not fixed constant as they had been previously but are allowed to change until the lowest mean square for the regression is obtained. As a result of these changes there is a broad variation in derived parameters: a, b, c, P_0_. Variations in the parameters were so great from one muscle to another that despite individual muscle trends and obvious differences in means there is no statistical difference from one condition to another when group data are evaluated. This method of analysis has only been used once previously by Donald et al. (1972) and they also found a great variability in the parameters obtained. Their range of a/P_0_ ratios was from 0.12 to 3.4 × 10^4^ concurring with the results of the present study.

Donald et al. (1972) also found that the predicted P_0_ from Eq. 1 or Eq. 1A was greater than the observed P_0_. The observed drop in velocity was sufficiently great in some reported instances that only the upper 2/3 of the curve was used when data were analyzed (Devrome and MacIntosh, 19852007; Gregor et al., 1988; Edman, 1993). In other cases, the authors appeared to have ignored the graphic discrepancy between their data and the predicted curve and accepted the statistical fit of the Hill model (Ritchie, 1954; Günther et al., 2018). When we employed this technique in the present study, the statistical fit of the data to Eq. 1A was good, but velocity at high loads dropped more rapidly than predicted. To correct this we devised a 5-parameter equation, Equation 2, which better accounted for the abrupt decrease in velocity at high loads (see Figure 12).

$$\mathrm{V=b}\left(\mathrm{c-P}\right)/\left(\mathrm{P+a}\right)+d\left(\mathrm{1-}e^{\left(f\left(\mathrm{P-c}\right)\right)}\right)$$ (2)

This is not the first alternative to the Hill equation that has been proposed. Alternatives were first espoused by Fenn and Marsh (1935), Donald et al. (1972) and Perreault et al. (2003) tried several different models among which were polynomials of different degrees, exponentials or a combination of exponentials, fourier series etc.

**FIGURE 13:**
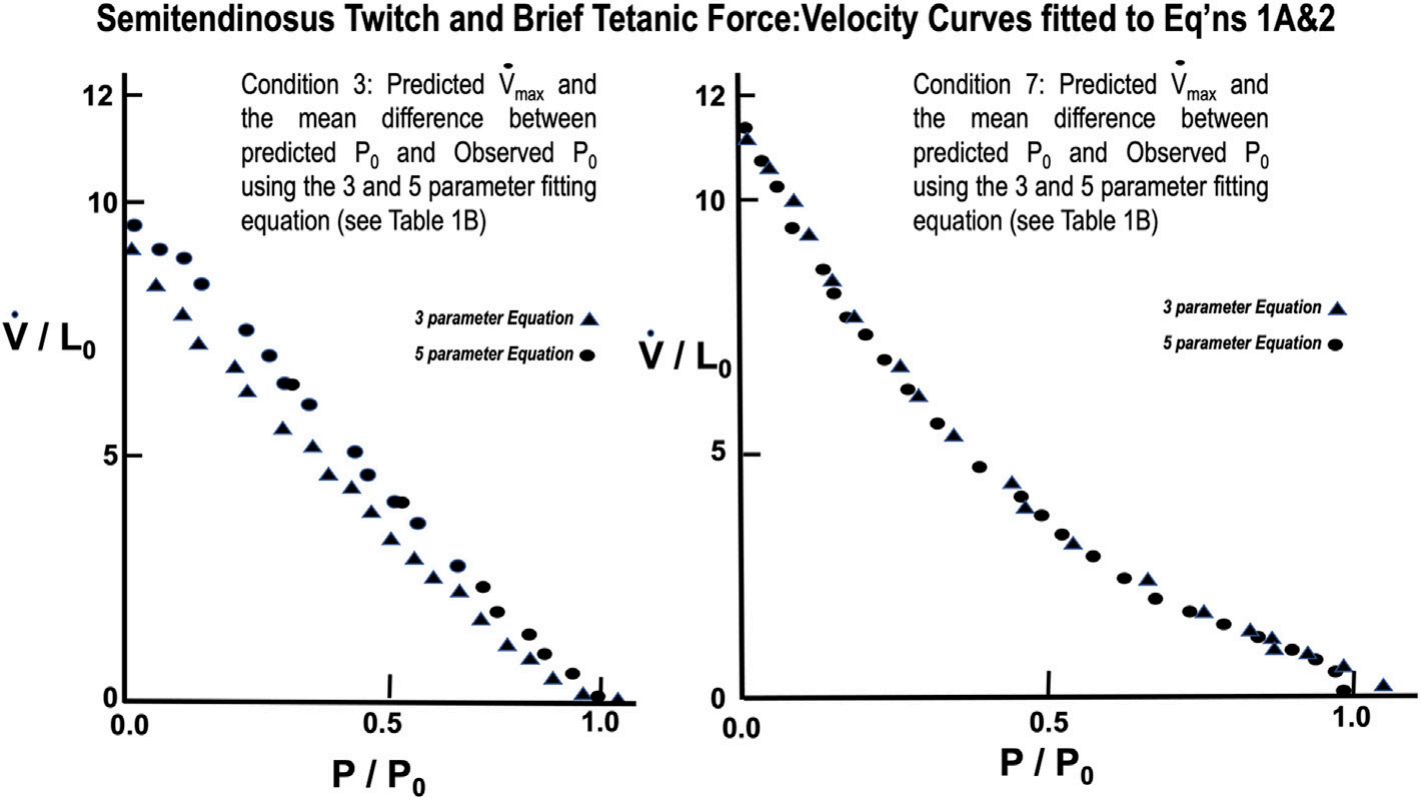
Force Velocity curves of observed data from semitendinosus with Twitch (L) and Brief Tetanic (R) stimulations fitted to Eqs 1A, 2. Comparison of the fit for the two equations was significantly better for Eq. 2 (*p* < 0.05).

From Table 1 lowering rest length and or reducing stimulus voltage tended to increase the a/P_0_ ratio, especially at initial lengths below 0.8 L_0_. Extrapolated V_max_ was also significantly reduced at rest lengths below 0.8 L_0_. This indicates a length dependent factor in the force velocity relationship. In all cases (twitch or brief tetanic), the percentage reduction of developed tension after reducing initial length and or stimulus voltage (up to 50%) is far greater than the reduction in extrapolated V_max_ (up to 15%). It is visually apparent for most of the curves that the five-parameter curve with its downward inflection, near P/P_0_ = 1 fits the observed data better than the simple rectangular hyperbola. (Figure 13) When compared statistically as a group, 14 experimental semitendinosus brief tetanic force:velocity curves had significantly better fits to the five than to the three-parameter equation due to the five-parameter equation’s ability to predict P_0_ better than the three-parameter equation. The difference between the observed and predicted P_0_ was 0.004 for the five-parameter equation compared to a mean difference of 0.088 for the three-parameter equation (*p* < 0.05).

**FIGURE 14:**
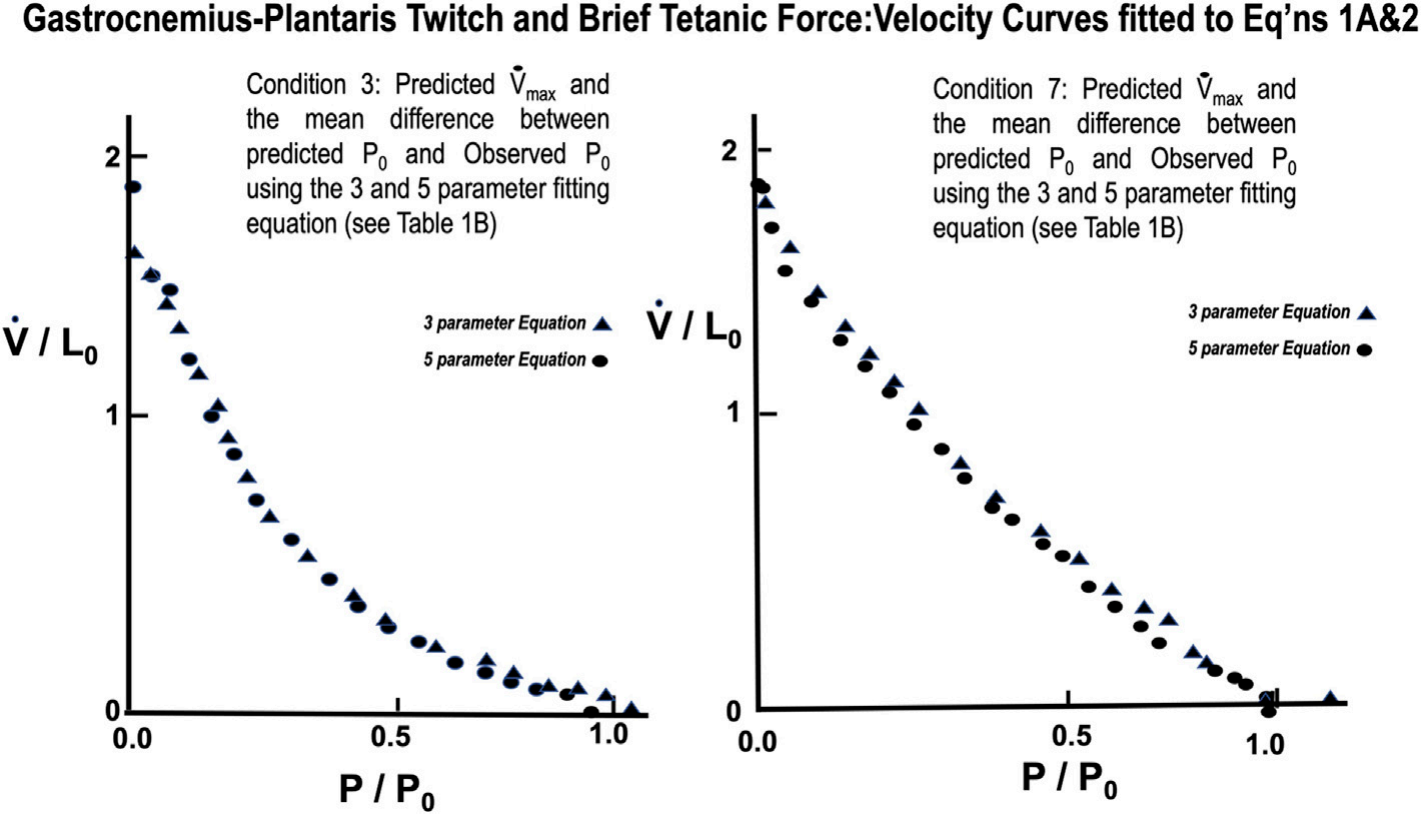
Force Velocity curves of observed data from gastrocnemius-plantaris with Twitch (L) and Brief Tetanic (R) stimulations fitted to Eqs 1A, 2. Comparison of the fit for the two equations was significantly better for Eq. 2 (*p* < 0.05).

The mean values for the derived parameters from curve fitting for the three-parameter and five-parameter equations for each stimulus condition for gastrocnemius plantaris are seen in Table 1. Fitted curves for Eqs 1A,2 for conditions 3 and 7 are seen in Figure 14. The five-parameter equation had a statistically better fit the observed data for the gastrocnemius plantaris than the three-parameter equation (*p* < 0.05). The difference was again especially exemplified by the differences between observed and predicted P_0_. The main difference between observed and predicted P_0_ for the five-parameter curve model was 0.012 compared to a mean difference of 0.061 for the three-parameter model (*p* < 0.05). Equation 2 is unquestionably necessary to describe the force:velocity relationship when velocity is carried through zero, to negative velocities (lengthening). Mashima et al. (1972) using frog skeletal muscle fiber bundles and Joyce and Rack (1969) using *in situ* cat soleus have measured the relationship of force-lengthening as well as force-shortening (Rack and Westbury, 1969). Their curves are in fact a double curve like ours, the upper half of which is hyperbolic and the lower half a negative exponential. This similar to what we found. The force component of computer fitted curves was carried through zero and velocity allowed to be negative. In all cases the transition occurs near zero velocity. To further justify the 5-parameter equation by calculating the velocity at tensions greater than P_0_ the parameters obtained from the regression after fitting Eqs. 3, 4 to a single set of observations. Five examples were carried out and the results were qualitatively the same in all cases.

Close, in his review (Close, 1972), stressed the importance of using units fiber lengths per second or sarcomere lengths per second rather than muscle lengths per second for expressing the velocity of muscle contraction. This is especially important if comparisons of different muscles are to be done. This importance of using the proper units is reemphasized in the present study when comparisons were done between semitendinosus and gastrocnemius plantaris. When muscle lengths per second are used the mean predicted maximal velocity for gastrocnemius plantaris is 1.97 compared to a mean of 9.61 for the semitendinosus. This would suggest that the gastrocnemius plantaris is much slower muscle than the semitendinosus. A major difference in speed between the two muscles would be unexpected in the light of observed similarities in the fiber type composition. When the two muscles maximum velocities were calculated using estimated fiber length rather than muscle length, the velocities were not significantly different, 7.88 for gastrocnemius plantaris versus 9.61 for the semitendinosus. Unfortunately, because it is really not possible to measure exact fiber length of every fiber in an intact pennate muscle, even with modern monitoring techniques, the estimated fiber length we used here is probably as close as can be measured. Analysis of the small difference between the two muscles that does exist leads to the hypothesis that fiber arrangement in the gastrocnemius plantaris is designed for strength rather than for speed. This hypothesis is supported by Azizi et al. (2008) who suggest that the pennation angle acts with the load acting like an automatic transmission system allowing the muscle to shift from high gear with low load allowing high velocity contractions to low gear as the load increases where forceful contractions are needed. However this view is somewhat controversial as Lieber suggests that muscle pennation has little if any functional significance (Lieber, 2022).

### Stress:Strain—Series Elastic Component

The stress: strain properties of the series elastic component using the standard quick release method of Wilkie (1949) were described in the methods section. The records obtained demonstrated a two-phase velocity record following release which concurs with the findings of previous studies (Hill, 1950; Bahler, 1967; Lieber et al., 2017). At L_0_, full stimulus voltage, the gastrocnemius plantaris series elastic component has a slightly greater compliance than the semitendinosus. Some of the reason for the difference in compliance between the two muscles may be due to the parallel elastic component, which must be stretched in the gastrocnemius to achieve L_0_. The effects of adding the parallel elastic to the series elastic would be to add the energy from the parallel elastic to the overall energy for shortening after the quick release. It is possible that the contractile element could also add its energy to the initial phase of shortening but it is assumed that it does not travel fast enough to play a significant role. The parallel elastic will only be a factor here if the muscle was stretched beyond L_0_ and would release all its energy by the time the muscle had reached L_0_ while shortening. However, the result of the addition of parallel elastic energy to the series elastic component would be an overestimation of the true series elastic. A second difference between the two muscles which may affect the series elastic is the quantity of series tendon within the two muscles. In the gastrocnemius plantaris the short muscle fibers are attached obliquely the parallel tendons which run through the muscle and a large thick tendon at the muscle clamp end of the muscle. These tendons may also add to the contractile element series elastic. The semitendinosus only has one thin tendonous inscription internally and a thin tendon at the muscle clamp end of the muscle. Thus, overall parallel tendon mass is probably less in semitendinosus than in gastrocnemius-plantaris. The combination of effects from parallel elastic and tendon probably accounts for the difference between the compliance values of the two muscles series elastic component at L_0_ full stimulus voltage.

Deliberately reducing muscle contractility by lowering stimulus voltage or rest length had no significant effect on the compliance of the serious elastic component in either muscle. However, when voltage and rest length were lowered simultaneously, there was an increase in the compliance of the serious elastic component in both muscles. Some of the possible explanations are as follows: (1) reducing stimulus voltage leaves some motor units completely inactivated, (2) reducing initial length is thought to reduce activation, and (3) there is a difference in the strength among different sarcomeres (Gordon et al., 1966; Rack and Westbury, 1969; Donald et al., 1972; Lambert et al., 1979; Allinger et al., 1996; Hou, 2018). Hill (1950) has proposed that inactivated or weak fibers will function as series elastic when the muscle contracts. Although, none of the differences for contractile state cause a significant increase and the series elastic separately, in combination the inactivated fibers and weak fibers could act together and significantly increase the total muscle serious elastic. This, however, is speculation and the actual mechanism for this increase in compliance is not certain.

The linear stress: strain curve of the series elastic component from here does not agree with most of the reported findings of previous investigators. Most previous mechanical studies on skeletal and cardiac muscle have reported that the stress: strain curve of the elastic component is defined by an exponential function (Hill, 1950; Pennycuick, 1964; Parmley et al., 1970; Sonnenblick and Skelton, 1974; Herbert and Gandevia, 19852019; Mayfield et al., 2016; Herzog, 2017; Lieber et al., 2017; Lieber and Binder-Markey, 2021). From reviewing the results of these investigators, the following relationship can be drawn concerning the differences between their data and the data from this study: (1) They were done at lower temperatures (0°C–4°C). Because the bend of the exponential function (the point of most rapid slope change) occurs at relatively low loads while at higher temperatures (17°C–25°C) the bend occurs at higher loads. Jewell and Wilkie (1958) and Yeatman et al. (1969) compared the stress strain curves from the same muscle at increasing temperatures and found the shape of the curve was altered significantly as temperature increased in the manner described above. If this effect can be extrapolated to 37°C perhaps the bend in the exponential would occur beyond P/P_0_ = 1 and therefore the curve would appear linear over the range of loads studied in this experiment. (2) Some of the previously reported stress strain curves are nearly linear and it appears they would fit either a linear or exponential model equally well (Ritchie, 1954, Donald et al., 1972). The majority of these studies were performed between 32^o^C and 37°C and the choice of an exponential may have just been due to the bias of the author (Joyce and Rack, 1969). Over damping of the signals may have distorted the data from the experimental quick release records presented by other authors. Almost, if not all the reported data from these studies demonstrate a much lower rate of oscillation in the length records after the quick release than is seen here (Hill, 1950; Pennycuick, 1964; Parmley et al., 1970; Sonnenblick and Skelton, 1974; Herbert and Gandevia, 19852019; Mayfield et al., 2016; Herzog, 2017; Lieber et al., 2017; Lieber and Binder-Markey, 2021). The slower oscillations may indicate a difference in the degree of damping and/or greater inertia in their system compared to the system used in the present study. The increased damping could have arisen from either increased damping in the muscle caused by the increased viscosity which occurs when the temperature is lowered, by over damping the lever or damping induced by the recording apparatus itself. Both latter possibilities could cause a linear relationship to become non-linear especially as change in load and length increases.

A final possibility for the difference is that the lever system used for this study was different than the ones used in previous mechanical studies because it used an air-spring lever without significant viscous damping (Fales et al., 1958). The inertia of our lever was only 1.9 g per Gram of muscle, a value much less than the relative inertia of lever systems employed in previous experiments e.g., Bahler (1967) lever had an inertial load of 6 g per Gram of muscle. Our studies measuring the compliance of simple springs by both quick release and static measurements confirmed the accuracy of the lever system and have shown our lever system is minimally damped. When quick releases from simple springs were compared to a quick release of the muscle there is no doubt that the serious elastic component is significantly damped by the muscle. The release curves from spring releases had a very high resonant frequency. However, if the springs were deliberately damped by manually holding the spring, the resulting results resembled actual muscle releases. Bahler (1967) found the damping of the series elastic component to be 300 dyn per square centimeter at 17.5°C. Thus, muscle viscosity must be responsible for some, if not all, of the damping of the series elastic component and would certainly be demperature dependent. A report by Wise et al. (1973) has shown that the stress:strain curve from glycerinated rabbit fibers to be either linear or to bend in the opposite direction from previously reported data. The records they present demonstrate oscillations which are similar in frequency to those we found here. This supports the data from our study and encourages further investigation to prove what part of the measurement is really from the muscle and what is only an equipment or temperature artifact.

## Conclusion

We have defined the mechanical properties of two blood perfused nerve stimulated canine skeletal muscles at normal body temperature. The differences that we observed related to differences in fiber arrangement between the long parallel fiber semitendinosus and the short pennate fiber gastrocnemius plantaris that were seen were for the most part quantitative and not qualitative. These differences include the sarcomere fiber overlap at L_r_ which was greater in the gastrocnemius, and required more passive tension to reach both L_i_ and L_0_. Its force velocity relationship was similar but based on muscle lengths the peak velocity was much lower, and it remained slightly lower even when estimates of velocity based on fiber lengths were made. Lastly the series elastic was significantly larger in gastrocnemius-plantaris, suggesting that there were more fibers per Gram of muscle, or that the connective tissue played a greater role in the measurements of series elastic than in semitendinosus. When compared to other studies on mammalian muscle at different temperatures, the major difference from most of them was that the series elastic component stress:strain relationship was linear and not exponential. One suggestion for that is that at temperatures lower than normal mammalian body temperature that the quick release recordings were damped either by the muscle itself or in the devices which recorded it giving it a non-linear relationship.

## Data Availability

The raw data supporting the conclusion of this article will be made available by the authors, without undue reservation.
